# Long-term intake of Tamogi-take mushroom (*Pleurotus cornucopiae*) mitigates age-related cardiovascular dysfunction and extends healthy life expectancy

**DOI:** 10.1038/s41514-024-00191-z

**Published:** 2025-01-08

**Authors:** Michio Sato, Daisuke Torigoe, Yuya Kinoshita, Momoka Cyuman, Chitoku Toda, Masaru Sato, Kazutaka Ikeda, Tsuyoshi Kadomatsu, Haruki Horiguchi, Jun Morinaga, Hirotaka Fukami, Taichi Sugizaki, Keishi Miyata, Ryoko Kusaba, Yusuke Okadome, Eiji Matsunaga, Koichi Node, Yuichi Oike

**Affiliations:** 1 Department of Molecular Genetics, Kumamoto University, Kumamoto, Japan; 2 Center for Metabolic Regulation of Healthy Aging (CMHA), Graduate School of Medical Sciences, Kumamoto University, Kumamoto, Japan; 3 Department of Cardiovascular Medicine, School of Medicine, Saga University, Saga, Japan; 4 Division of Kumamoto Mouse Clinic (KMC), Kumamoto University, Kumamoto, Japan; 5 Division of Laboratory Animal Science, Institute of Resource Development and Analysis (IRDA), Kumamoto University, Kumamoto, Japan; 6 Department of Neuroscience for Metabolic Control, Graduate School of Medical Sciences, Kumamoto University, Kumamoto, Japan; 7 Laboratory of Biomolecule Analysis, Department of Applied Genomics, Kazusa DNA Research Institute, Chiba, Japan; 8 Laboratory of Omics and Informatics, Department of Molecular and Chemical Life Sciences, Graduate School of Life Sciences, Tohoku University, Sendai, Japan; 9 Department of Aging and Geriatric Medicine, Graduate School of Medical Sciences, Kumamoto University, Kumamoto, Japan; 10 Department of Disease Genome Epidemiology, Center for Genomic Medicine, Graduate School of Medicine, Kyoto University, Kumamoto, Japan

**Keywords:** Cardiovascular diseases, Ageing

## Abstract

Age-related declines in cardiac function and exercise tolerance interfere with healthy living and decrease healthy life expectancy in older individuals. Tamogi-take mushrooms (*Pleurotus cornucopiae*) are known to contain high levels of Ergothioneine (EGT), an antioxidant with potential health benefits. In this study, we assessed the possibility that long-term consumption of Tamogi-take mushrooms might attenuate age-related decline in cardiac and vascular endothelial function in mice. We found that long-term intake of Tamogi-take mushrooms significantly maintained cardiac and vascular endothelial function and improved exercise tolerance in mice. Long-term mushroom consumption also increased levels of Nrf2 (Nuclear factor E2-related factor 2) protein in heart tissues and increased translation of HO-1 (Heme Oxygenase 1) proteins, which have antioxidant effects in heart and aortic tissues. Finally, long-term Tamogi-take mushroom consumption inhibited ROS accumulation with aging and reduced expression of inflammatory biomarkers. We conclude that ingestion of Tamogi-take mushrooms could serve as a dietary intervention to promote cardiovascular health, support healthy aging and slow the progression of age-related diseases.

## Introduction

Due to worldwide increases in human life expectancy^1^, many countries are rapidly becoming aging societies^2^. In particular, the proportion of aged individuals in Japan is extremely high relative to other countries, resulting in a potentially “super-aging” society^3–5^ Specifically, in Japan there is currently a gap of ~10 years between healthy life expectancy and average life expectancy, and shortening this gap to promote healthy aging remains a challenge^6,7^.

Based on recent reports in Japan, age-related frailty has overtaken cerebrovascular disease and dementia (previously, the two leading health concerns among the aging) as the leading factor in reducing healthy life expectancy^8–10^. Age-related functional decline in various organs in the body may underlie the onset of frailty^11,12^. Given that life begins and ends with the respective onset and cessation of a heartbeat, we focused on the critical importance of maintaining normal cardiac function over an individual’s lifetime. Moreover, we recently reported that centenarians die within a few years of a decline in cardiac function, supporting the idea that cardiac decline shortens maximum life expectancy^13^. Even in non-elderly individuals with normal locomotion and healthy muscle and bone function, a sudden decline in cardiac function and subsequent heart failure can promote exercise intolerance and progressively limit activity^14–16^. These findings suggest that counteracting declines in cardiac function may counteract exercise intolerance, prevent development of age-related frailty, and extend a healthy life span^17,18^.

Factors contributing to the age-related decline in cardiac function include mitochondrial dysfunction^19^, genomic instability, epigenetic changes, autophagy dysfunction, and chronic inflammation^20^, all recognized as hallmarks of aging^21^. Among these hallmarks, reactive oxygen species (ROS) play a particularly important role^22,23^. Controlled generation of free radicals, ROS and other reactive species is a necessary consequence of aerobic metabolism^24^ and is required to antagonize infection and other disorders^25^. However, ROS and other reactive species react with biopolymers such as DNA, lipids, proteins, and enzymes in vivo, resulting in lipid peroxidation, DNA mutation, protein denaturation, and enzyme inactivation^26^. Aging-related oxidative stress promotes cellular senescence, triggering the senescence-associated secretory phenotype (SASP), which promotes chronic inflammation and tissue dysfunction, both drivers of cardiovascular decline and other age-related diseases^27^. Accordingly, interventions to block these activities may prevent or slow cardiovascular aging^28–32^.

Ergothioneine (EGT), a naturally derived antioxidant with potent antioxidant properties, was isolated and discovered in 1909 by M.C. Tanret in rye horn fungus (scientific name: *Claviceps purpurea*)^33^. Other mushrooms and dietary sources^34^ also contain EGT^35^, which have garnered attention for potential health benefits^36,37^. Tamogi-take mushrooms are known to contain particularly high levels of EGT^38^.

Recent studies indicate that EGT exhibits potent antioxidant and cytoprotective properties by scavenging free radicals and protecting cells from oxidative stress-induced damage^39,40^. Furthermore, EGT’s unique OCTN-1-mediated transport mechanism allows efficient uptake and distribution to various organs, and a wide range of organ-protective effects have been reported^41–45^.

However, the specific effects of long-term consumption of Tamogi-take mushrooms on cardiac and vascular functions related to aging remain unknown.

This study addresses these questions by evaluating cardiac and vascular endothelial function in mice fed a long-term diet of Tamogi-take mushrooms. Here, we show that long-term daily intake of Tamogi-take mushrooms prevents age-related cardiovascular dysfunction, resulting in improved exercise tolerance. We also show that this effect is mediated by activation of the Nrf2-HO-1 pathway, which blocks ROS accumulation in the heart and vascular endothelium. This work has implications not only for dietary interventions but also for understanding mechanisms underlying age-related cardiovascular changes and developing targeted strategies to promote healthy aging.

## Results

### Mice fed a mixture of chow plus Tamogi-take mushrooms decrease their food intake

**Fig. 1:**
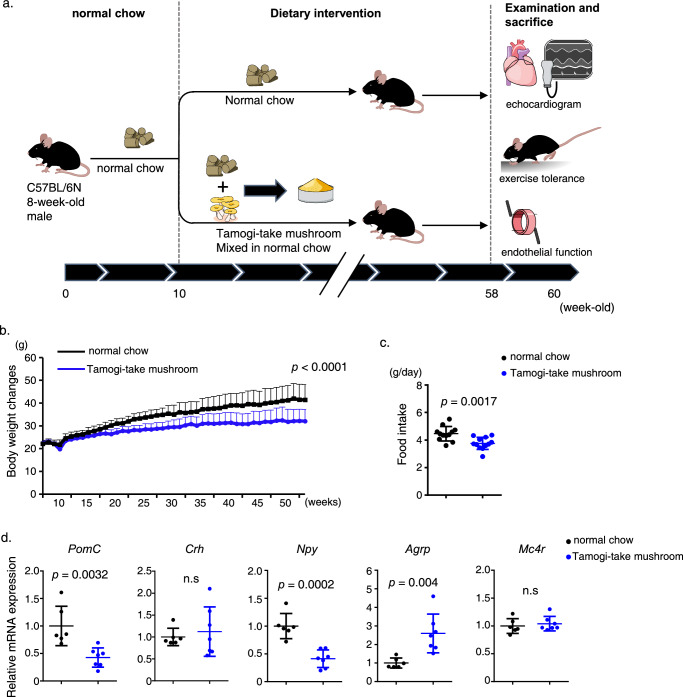
Mice fed a mixture of Tamogi-take mushrooms and chow show decreased food intake. **a** Experimental design. **b** Body weight over time (normal chow: *n* = 10, Tamogi-take mushroom: *n* = 11 at start). One animal from each group died during the analysis. **c** Food intake per day (*n* = 12 per group). **d** Relative expression of genes associated with appetite regulation in hypothalamus tissue of indicated mice (normal chow, *n* = 6; Tamogi-take mushrooms, *n* = 7). Levels seen in mice fed normal chow were set to 1. Each data point represents a mouse. All data are presented as the mean ± SD. *P* < 0.05 indicates statistical significance. n.s. not significant. Data were analyzed using a two-sided unpaired Student’s *t* test.

Initially, we divided 8-week-old male C57BL/6N mice into two groups: one fed powdered normal chow (CE2) mixed with heated, dried and powdered Tamogi-take mushrooms and administered ad libitum, and the other (control) group fed normal chow only (Fig. 1a). We adjusted the dose of Tamogi-take mushrooms weekly to provide the equivalent of 70 mg/kg/day of EGT (Supplementary Fig. 1). We observed that the group fed the mushroom/chow mix gained weight more slowly than did controls, and when animals were analyzed 12 months later, that group weighed significantly less than the control group (Fig. 1b). Thus, to assess levels of food intake in each group, we again divided 8-week-old mice into the same two groups and observed their food intake over a four-day period. That analysis indicated that daily food intake was approximately 15.8% lower in the group fed the mushroom/chow mix versus the normal chow group (Fig. 1c). To confirm that feeding mice Tamogi-take mushroom/chow mix decreases their food intake, we divided 8-week-old C57BL/6N male mice into two groups in which one group was initially fed the mushroom/chow mix for several days before being switched to normal chow, while a second group was initially fed normal chow for several days before transitioning to the mushroom/chow mix. That analysis indicated that food consumption decreased during periods when both groups were fed the Tamogi-take mushroom/chow mixture (Supplementary Fig. 2). Next, we evaluated the expression of appetite-regulating genes in the hypothalamus of 10-week-old male mice fed either the mushroom/chow mix or normal chow over 2 weeks. Consumption of Tamogi-take mushrooms did not alter the expression of appetite-regulating genes in a consistent manner (Fig. 1d), suggesting that mice may not prefer Tamogi-take mushrooms, and there was a 22.1% weight difference between the two groups at the time of cardiac and vascular function assessment. However, our study design did not include a fasting period which some report to be an important factor in calorie restriction (CR), a practice shown useful in improving health.

### Long-term feeding with Tamogi-take mushrooms attenuates age-related cardiac dysfunction and improves exercise tolerance in mice

**Fig. 2:**
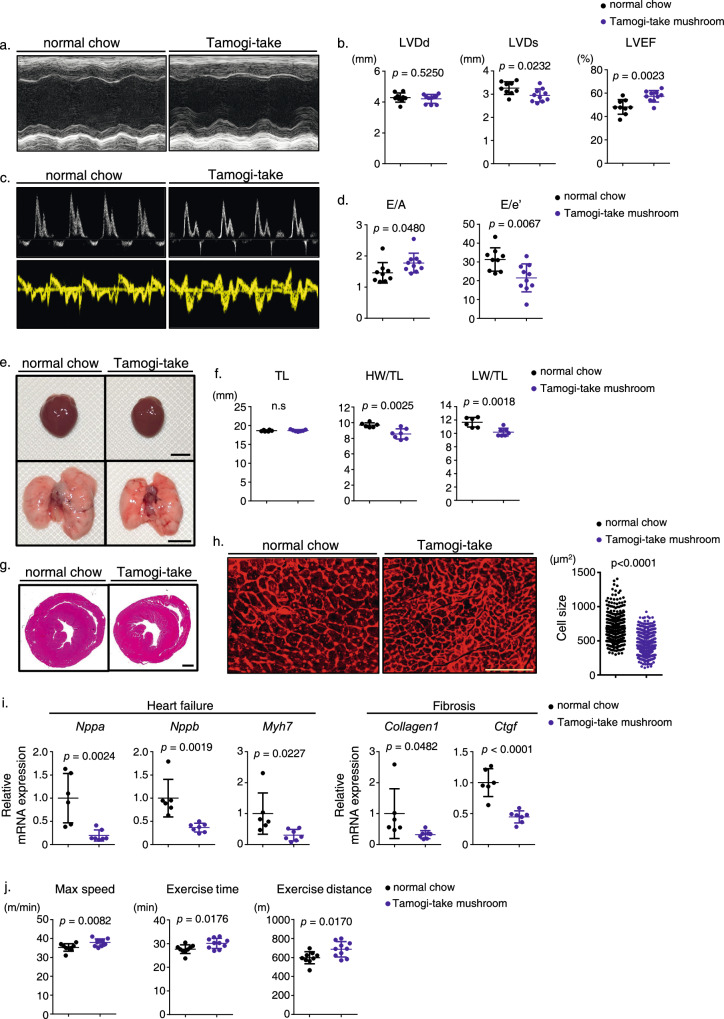
Long-term Tamogi-take mushroom feeding attenuates age-related cardiac dysfunction and improves exercise tolerance in mice. **a** Representative M mode images of echocardiography. **b** Left ventricular end-diastolic diameter (LVDd) (mm) (left), left ventricular end-systolic diameter (LVDs) (mm) (middle), and left ventricular ejection fraction (LVEF) (right) in indicated mice (*n* = 9–10 per group). **c** Representative Pulse Wave (PW) echocardiography of Mitral valve inflow (upper) and Tissue Doppler (lower) recordings from indicated mice. **d** E/a (left) and E/e’ (right) ratios in indicated groups (*n* = 9–10 per group). **e** Gross appearance of whole heart (upper; scale bar, 5 mm), gross appearance of whole lung (lower; scale bar, 5 mm). **f** Body weight (BW) (g), Tibia Length (TL) (mm), Heart weight per Tibia length ratio (mg/mm), and lung weight/ tibia length (LW/TL) ratio (mg/mm) in indicated mice (*n* = 6–7 per group). **g** Hematoxylin–eosin (HE)-stained sections of the mid-portion of the heart (scale bar, 1 mm). **h** Representative sections of left ventricle stained with wheat germ agglutinin (WGA) to indicate cardiomyocyte size in groups fed normal chow or the Tamogi-take mushroom mixture (left). Size distribution of myocardial cells (μm^2^) in indicated mice (right); the number of cells = 354 (normal chow) and 370 (Tamogi-take mushroom). Scale bar, 100 μm. **i** Relative expression of genes associated with heart failure and fibrosis in hearts of indicated mice (*n* = 6–7 per group). **j** Exercise tolerance test showing max speed (m/min) (left), exercise time (min) (middle), and exercise distance (m) on a treadmill test (*n* = 9–10 per group). Levels seen in mice fed normal chow were set at 1. Plots for (**b**, **d**, **f**, **h**–**j**) present all data points, median and ± SD. Statistical significance was determined by a two-sided unpaired Student’s *t* test.

We next asked whether consumption of Tamogi-take mushrooms conferred cardioprotective effects and mitigated age-related deterioration in cardiac function. To do so we fed mice for 12 months with either the control chow diet or a mix of Tamogi-take mushrooms plus chow and assessed cardiac function by echocardiography. That analysis indicated that mice receiving Tamogi-take mushrooms exhibited improved age-associated cardiac left ventricular systolic function (Fig. 2a, b) and reduced dilatation dysfunction relative to control mice (Fig. 2c, d). Furthermore, while aging and heart failure typically induce myocardial hypertrophy, this hypertrophic response was ameliorated in mice supplemented with Tamogi-take mushrooms (Fig. 2e). Heart Weight to Anterior Tibial length (HW/TL) and Lung Weight to Anterior Tibial length (LW/TL) ratios also decreased in mice fed Tamogi-take mushrooms relative to controls (Fig. 2f). Histological analysis revealed a reduction in cardiomyocyte enlargement in mice fed mushrooms versus controls (Fig. 2g, h), and RT-PCR analysis of heart tissue revealed significantly reduced expression of genes associated with heart failure or cardiac fibrosis in mushroom-fed versus control mice (Fig. 2i). Treadmill test performance also significantly improved in 12-month-old (middle-aged) mice fed Tamogi-take mushrooms relative to controls (Fig. 2j and Supplementary Video). We observed no changes in blood pressure or pulse rate as a result of Tamogi-take mushroom feeding (Supplementary Fig. 3). These results indicate that long-term intake of Tamogi-take mushrooms ameliorated age-related cardiac decline and underlying changes in the heart and improved exercise tolerance capacity.

### Tamogi-take mushroom intake blocks ROS accumulation and upregulates Nrf2 and HO-1 protein levels in mouse heart

**Fig. 3:**
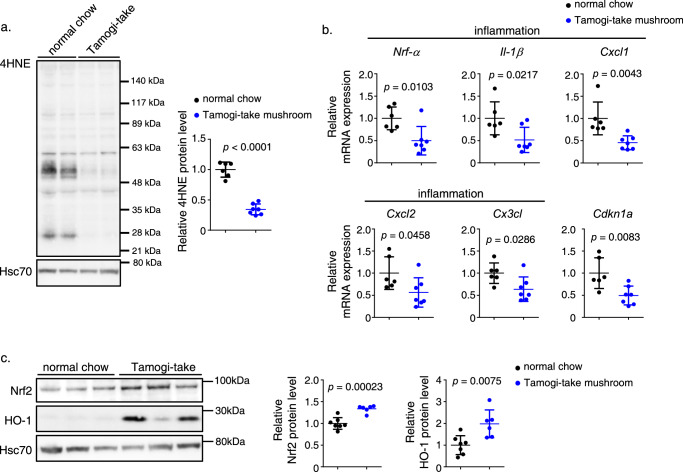
Ten months of feeding with Tamogi-take mushrooms blocks ROS accumulation and upregulates Nrf2 and HO-1 protein levels in mouse heart. **a** Representative western blots (left) and quantitation (right) of 4-HNE-modified protein staining in heart tissues of indicated groups (*n* = 6–7 per group). **b** Relative expression of genes associated with inflammation and senescence (*n* = 6–7 per group) in heart tissues from indicated groups. Values in the control group were set to 1. **c** Representative western blots (left) and quantitation (right) of Nrf2 (upper) and HO-1 protein (lower) staining in heart tissues of indicated groups (*n* = 6–7 per group). In (**a**, **c**), Hsc70 served as loading control. Values in the control group were set to 1. Plots for (**a**–**c**) present all data points, median and ± SD. Statistical significance was determined by a two-sided unpaired Student’s *t* test.

To define potential heart-protective mechanisms promoted by Tamogi-take mushroom intake we assessed ROS accumulation and EGT-related antioxidant pathways in hearts of mice fed 12 months with either Tamogi-take mushroom/chow mix or control chow only. Immunohistochemical analysis of lipid peroxidation in heart tissues from each group indicated higher 4HNE levels in tissues from control versus mushroom-fed mice, indicative of greater ROS accumulation in those control samples (Fig. 3a). Furthermore, levels of biomarkers of inflammation and senescence were lower in heart tissues from mice fed Tamogi-take mushrooms versus controls (Fig. 3b). Moreover, we observed increased levels of Nrf2 and HO-1 proteins in heart tissues from mice fed mushrooms versus controls (Fig. 3c). HO-1 protein has antioxidant properties, while Nrf2 is a transcription factor functioning in promoting cellular antioxidant responses and the gene expression of cell survival, mitochondrial function maintenance, and inflammation suppression, thereby protecting cells from damage and promoting tissue resilience. EGT reportedly increases transcription of the gene encoding HO-1, which in turn prevents Nrf2 binding to the adaptor protein Keap1, thereby preventing proteasomal Nrf2 degradation in dermatological study^46,47^. Our analysis indicates that this previously reported mechanism may extend to the heart and suggests that the antioxidant effects of Tamogi-take mushrooms are due to the effects of EGT.

### Long-term feeding with Tamogi-take mushrooms attenuates age-related vascular endothelial dysfunction in mice

**Fig. 4:**
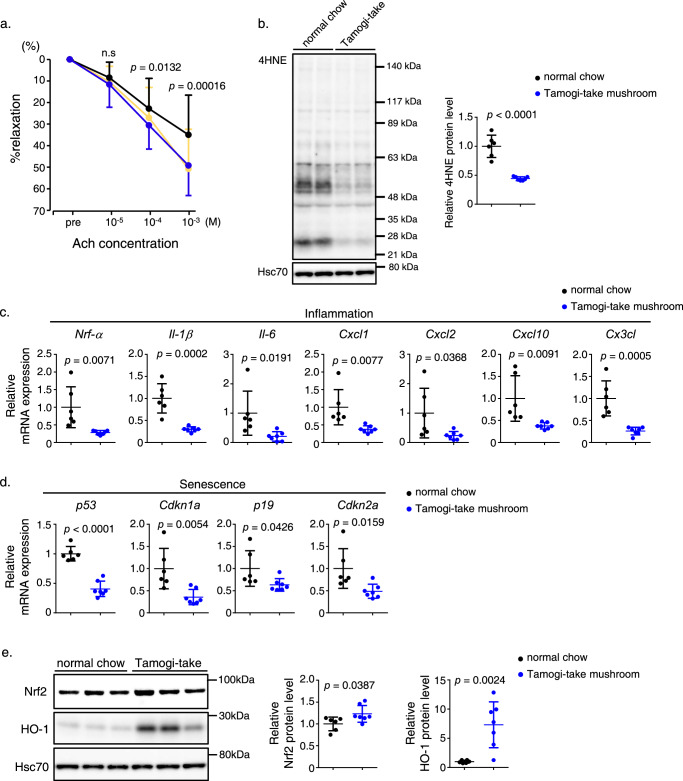
Long-term feeding of Tamogi-take mushrooms attenuates age-related vascular endothelial dysfunction in mice. **a** Vasorelaxation responses (%) ex vivo to acetylcholine (Ach) (*n* = 9–11 per group). **b** Representative western blots (left) and quantitation (right) of 4-HNE-modified protein staining in aorta tissues of indicated groups (*n* = 6–7 per group). **c**, **d** Relative expression of genes associated with inflammation (**c**) and senescence (**d**) in aorta tissues from indicated groups (*n* = 6–7 per group). Values in the control group were set to 1. **e** Representative western blots (left) and quantitation (right) of Nrf2 (upper) and HO-1 protein (lower) staining in aorta tissues of indicated groups (*n* = 6–7 per group). In band (**d**), Hsc70 served as a loading control. Values in the control group were set to 1. Plots for (**b**–**e**) present all data points, median and ± SD. Statistical significance was determined by a two-sided unpaired Student’s *t* test.

Next, we asked whether Tamogi-take mushroom intake affected age-related vascular endothelial dysfunction. As shown in Fig. 4a, assays of vascular endothelial function using an organ chamber showed that the endothelium of 10-week-old young mice responded well to acetylcholine and showed sufficient relaxation after endothelin-induced contraction, while the 14-month-old normal chow group showed significantly impaired relaxation. On the other hand, mice fed Tamogi-take mushrooms at the same age showed almost no age-related decline in vascular endothelial function and maintained vascular endothelial function comparable to that of younger mice (Fig. 4a). In these mice, ROS accumulation was remarkably attenuated in the vascular endothelium of mushroom-fed compared to control mice (Fig. 4b). Assessment of inflammation and senescence biomarkers potentially upregulated by ROS accumulation showed a significant decrease in the mushroom-fed group relative to controls (Fig. 4c, d). Furthermore, we confirmed that these results were accompanied by increased Nrf2 and HO-1 protein levels in the vascular endothelium of mice-fed Tamogi-take mushrooms, as in the heart (Fig. 4e).

### Tamogi-take mushrooms used in this study contain high levels of Ergothioneine

Tamogi-take mushrooms reportedly contain high levels of EGT (structure shown in Supplementary Fig. 4a). To confirm this, we performed LC-MS/MS analysis to assess EGT levels in mushrooms (sourced from Athree Inc., Kumamoto, Japan) used in this analysis (Supplementary Fig. 4b, c). Liquid chromatography analysis detected 7.6 mg EGT/g mushrooms; a value significantly higher than levels found in other foods reported previously^48^ (Supplementary Fig. 4d).

### Cardiovascular protective effects of Tamogi-take mushrooms are due to mushroom intake, not to calorie restriction

**Fig. 5:**
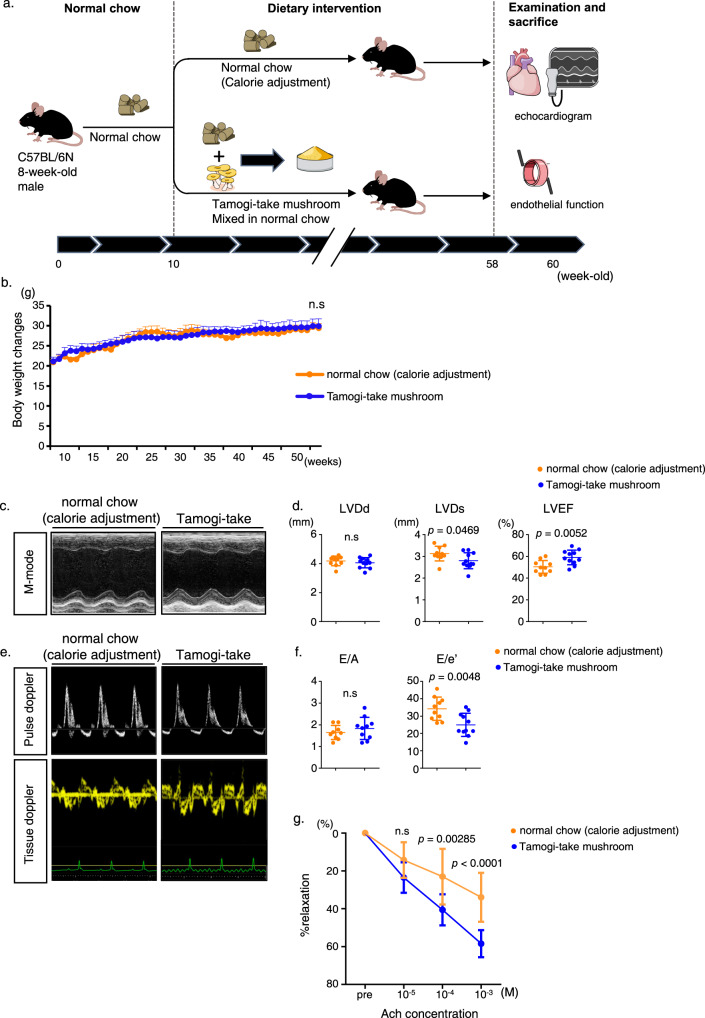
Tamogi-take mushroom-fed mice subjected to mild CR show limited cardiovascular-protective effects. **a** Experimental design. **b** Body weight over time (normal chow: *n* = 10, Tamogi-take mushroom: *n* = 11). **c** Representative M mode images of echocardiography. **d** Left ventricular end-diastolic diameter (LVDd) (mm) (left), left ventricular end-systolic diameter (LVDs) (mm) (middle), and left ventricular ejection fraction (LVEF) (right). **e** Representative Pulse Wave (PW) echocardiography of Mitral valve inflow (upper) and Tissue Doppler (lower) images. **f** E/A (left) and E/e’ (right) ratios in indicated groups (*n* = 10–11 per group). **g** Vasorelaxation responses (%) ex vivo to acetylcholine (Ach) (*n* = 8–10 per group). Plots for (**d**, **f**) present all data points, median and ± SD. Statistical significance was determined by a two-sided unpaired Student’s *t* test.

Evidence presented here suggests that long-term consumption of Tamogi-take mushrooms alleviates age-related cardiovascular deterioration due to EGT antioxidant effects. Nevertheless, we could not rule out the possibility that the cardioprotective effects seen in mushroom-fed mice were due to reduced food intake. To assess this possibility, we first divided 10-week-old C57BL/6N male mice into two groups—one reared on normal chow mixed with Tamogi-take mushrooms and the other fed normal chow with the dosage adjusted weekly to ensure that both groups exhibited comparable body weight. 12 months later, we assessed cardiac and vascular endothelial function (Fig. 5a, b). We observed that age-related left ventricular systolic function improved in mushroom-fed mice (Fig. 5c, d), while diastolic dysfunction was suppressed relative to mice that had received calorie-adjusted normal chow (Fig. 5e, f). Furthermore, vascular endothelial function was also preserved in the Tamogi-take-fed mice compared to the group receiving calorie-adjusted normal chow (Fig. 5g). No changes in blood pressure or pulse rate were observed in calorie-adjusted and Tamogi-take mushroom-fed groups (Supplementary Fig. 5). These results suggest that Tamogi-take mushroom intake decreases age-related cardiovascular decline and likely reflects the antioxidant potential of EGT.

## Discussion

This study investigates the potential benefits of long-term Tamogi-take mushroom intake on age-related cardiac and vascular dysfunction in mice, focusing on the antioxidant compound EGT present in these mushrooms. We report that Tamogi-take mushroom consumption significantly mitigates age-related cardiac dysfunction and vascular endothelial dysfunction. Specifically, mice undergoing 12 months of long-term feeding with Tamogi-take mushrooms exhibited notable preservation of cardiac function, as indicated by maintenance of contractile and diastolic capacities, suppression of heart failure and fibrosis marker expression, and inhibition of increased heart weight, suggestive of mitigation of age-related cardiac dysfunction. We also observed improved exercise tolerance in the mushroom intake group, indicating a potential delay in age-related decline in exercise capacity. In terms of vascular health, the long-term Tamogi-take mushroom intake group showed marked improvements in vascular endothelial function relative to controls, effects comparable to those seen in younger mice.

Specifically, our findings suggest that the cardioprotective effects of long-term intake of Tamogi-take mushrooms during aging are likely due to a reduction in ROS levels and concomitant SASP effects (Figs. 3a and 4b). We also showed that mushroom consumption significantly decreased levels of transcripts encoding the senescence markers p53, p21, and p16, and of inflammatory markers in the heart and vascular endothelium (Figs. 3b and 4c, d).

We propose that ROS suppression by mushroom consumption is likely due to EGT, a compound abundant in Tamogi-take mushrooms. These findings confirm previous studies showing that Tamogi-take mushrooms have antioxidant properties due to EGT content^40^. Indeed, here, we confirmed that Tamogi-take mushroom intake upregulates Nrf2 protein levels in the heart and vascular endothelium. Functionally, EGT likely activates Nrf2, increasing expression of antioxidant enzymes such as HO-1, which mitigate ROS-induced damage in tissues^49^. This finding is supported by in a vivo study on the effects of dietary EGT supplementation on oxidative damage in healthy young adults^50^.

Our study provides novel insights into the cardioprotective and vascular benefits of Tamogi-take mushroom intake as a part of the daily diet, particularly against aging-related dysfunction. Our detailed assessment of cardiac and vascular function in mushroom-fed and control mice confirms the significance of our findings.

We also asked whether cardioprotective phenotypes seen in mice fed Tamogi-take mushroom/chow long-term were due to reduced food intake and concomitant decreases in mouse BW. To do so, we compared cardiac and vascular endothelial function in mice fed the mushroom mix with that observed in mice fed only normal chow, whose food intake was adjusted so that the groups showed no change in body weight. In this experiment, calorie adjustment alone did not improve age-related cardiac or vascular endothelial function observed with Tamogi-take mushroom administration. Several reasons may underlie these effects. First, most previous studies reporting healthy longevity phenotypes generally employ 30% calorie restriction (CR)^51,52^. In our experiment, CR was ~18.6% compared to the free-feeding group. Recent studies also report the importance of a period of fasting in terms of CR effects^53,54^. In contrast, our experimental design did not include a time period in which mice were unable to feed. We conclude that the calorie adjustment protocol used in this study did not provide the cardiovascular protective effects previously associated with CR.

These findings suggest that prolonged consumption of Tamogi-take mushrooms may promote healthy longevity by mitigating age-related cardiovascular decline and preserving exercise tolerance, attributable to their antioxidant properties. Specifically, EGT abundance in Tamogi mushrooms is likely pivotal in these effects. EGT is resistant to heat and acid, remains stable during cooking^55^, and has a prolonged shelf-life, all qualities facilitating its daily consumption and underscoring its practical value.

Finally, although we attribute the beneficial effects in mice of Tamogi-take mushrooms on cardiovascular health primarily to their EGT content, it is possible that EGT supplementation alone could provide similar benefits. We note, however, that other bioactive compounds present in whole mushrooms may enhance EGT’s overall effectiveness, suggesting that mushrooms may be a preferable EGT source. Future research should investigate whether their consumption antagonizes cardiovascular aging in humans and assess potential longevity benefits, dosage optimization, and possible interactions with other dietary factors.

### Conclusion

Our study highlights Tamogi-take mushrooms as a promising dietary intervention to mitigate age-related cardiac and vascular dysfunction through antioxidant mechanisms involving EGT-induced Nrf2 activation.

## Methods

### Animal studies

All experimental procedures were approved by the Kumamoto University Ethics Review Committee for Animal Experimentation (approval No. A27-063, A29-072, A2019-063, A2021-071 and A2023-039). All animals were fed normal chow (ND; CE-2, CLEA, Tokyo, Japan), bred in a mouse house with automatically controlled lighting (12 h on, 12 h off), and maintained at a stable temperature of 22 ± 2 °C and a relative humidity of 40–80%. Tamogi-take mushrooms were a dried powder preparation provided by Athree Inc, and their intake was 9.3 g/kg/day, such that mushrooms were included in normal chow. This diet was calculated to be ~3.3% lower in calories than CE-2, assuming a daily intake of 4 g (Supplementary Table 1).

### Echocardiography

Mice were preconditioned by chest hair removal with a topical depilatory (FUJIFILM VisualSonics, Toronto, Canada), anesthetized with 1.5–2.5% isoflurane administered via inhalation, and maintained in a supine position on a dedicated animal handling platform with limbs attached for electrocardiogram gating during imaging. Body temperature was kept constant by feeding the signal of a rectal probe back to a heating pad, while heart and respiratory rates were continuously monitored. Transthoracic echocardiography was performed using a high-frequency ultrasound system dedicated to small animal imaging (VisualSonics Vevo 3100, FUJIFILM VisualSonics, Toronto, Canada) using a MS 400 linear array transducer (18–38 MHz). M-mode recording was performed at the midventricular level. All images were analyzed using dedicated software (Vevo Labo version 5.7.1). LV wall thickness and internal cavity diameters at diastole (LVID; d) and systole (LVID;s) were measured. Percent LV fractional shortening (%FS) was calculated from M-mode measurements. For analysis of cardiac diastolic function, transmitral flow velocity patterns and early diastolic mitral annular velocity (e’) were measured to calculate early transmitral flow velocity (E) to atrial systolic velocity (A) (E/A) and the ratio E to e’ (E/e’) in 4-chamber view. All procedures were performed under double-blind treatment conditions.

### Mouse endurance exercise testing

Experimental mice were allowed to adapt to the treadmill chamber (Model MK-690S/4 M, Muromachi, Japan) for 15 min with unlimited movement 3 times a day on separate days. After confirming that mice were sufficiently accustomed to running, we began the treadmill test at 5 m/min, and after 30 s, increased speed to 6 m/min. After the first minute, speed was increased to 8 m/min and then to 10 m/min after another minute. Speed was then increased by 1 m/min every minute. The experiment was designed to be terminated when a mouse touched the electrodes on ten or more occasions during rest intervals. The electrodes were positioned at the rear of the treadmill belt. The test was terminated for any mouse that made contact with the electrodes ten times while running at a constant speed.

### Ex vivo endothelium-dependent relaxation assay

Murine aortas of equal lengths (2.5 mm) were cut and mounted in a 4-ml organ chamber filled with Krebs-Ringer bicarbonate solution (118.4 mMol/L NaCl, 4.7 mMol/L KCl, 2.5 mMol/L CaCl_2_, 1.2 mMol/L KH_2_PO_4_, 1.2 mMol/L MgSO_4_, 25.0 mMol/L NaHCO_3_, 10.0 mMol/L glucose: 37 °C, pH 7.4), and bubbled with 95% O_2_, 5% CO2. Aortic rings were connected to an isometric force transducer (PowerLab 4/26 and LabChart v7.2, AD Instruments Japan, Inc. Nagoya, Japan.) for continuous isometric tension recording (Micro tissue organ bath,　Model MTOB-1Z, Labo Support, LLC, Osaka, Japan). Concentration–response curves were obtained in response to increasing acetylcholine (Ach) concentrations (1.0 × 10^−5^–1.0 × 10^−3^ Mol/L; TOKYO CHEMICAL INDUSTORY CO., LTD, Tokyo, Japan) in vessels pre-treated with phenylephrine at 1.0 × 10^−5^ Mol/L (TOKYO CHEMICAL INDUSTORY CO., LTD).

### RT-PCR analysis

Total RNA was extracted using a RNeasy Mini Kit (Qiagen, Valencia, CA, USA). DNase-treated RNA was reverse transcribed using a Prime Script RT reagent Kit (Takara Bio Inc, Shiga, Japan). Heart tissue was homogenized using a multi-beads shocker (Yasui Kikai, Osaka, Japan). Real-time quantitative RT-PCR was performed using TB Green Premix Ex Taq II, Premix Ex Taq (Probe qPCR) (Takara Bio Inc), and a Thermal Cycler Dice Real-Time system (Takara Bio Inc). Relative transcript abundance was normalized to that of *18S* rRNA levels in mouse samples. Primer sets used for RT-PCR are listed in Supplementary Table. 2.

### Western blotting

Mouse heart tissue was homogenized in RIPA buffer: 50 mM Tris–HCl (pH 7.0), 0.1 mM EDTA (pH 8.0), 0.5%DOC-Na, 1% NP-40, 0.1% SDS, 150 mM NaCl, 10 mM Na_4_P_2_2O_7_, 100 mM NaF, 0.1 mM Na_3_VO_4_, plus a protease inhibitor cocktail (Nacalai Tesque, Kyoto, Japan), pH 7.5 using a multi-beads shocker (Yasui Kikai). Proteins (20 mg) were separated by SDS–PAGE and transferred to PVDF membranes. Membranes were incubated with anti-4-hydroxy-2-nonenal (4-HNE) (clone HNEJ-2, MHN, Japan Institute for the Control of Aging (JaICA), NIKKEN SEIL, Shizuoka, Japan), anti-NRF2 (D1Z9C, #12721, Cell Signaling Technology, Danvers, MA, USA 12721) and anti-HO-1 (E3F4S, #43966, Cell Signaling Technology). Antibodies were diluted 1:1000, and samples were incubated at 4 °C overnight. After TBST washing, membranes were incubated with 1:2000 diluted horseradish peroxidase (HRP)-conjugated donkey anti-rabbit IgG or sheep anti-mouse IgG (GE Healthcare Life Science, Piscataway, NJ, USA) antibodies at room temperature for 60 min. Internal controls were incubated with 1:2000 diluted anti-Hsc70 (sc-7298, Santa Cruz Biotechnology, Santa Cruz, CA, USA) and 1:2000 diluted HRP-conjugated sheep anti-mouse IgG (GE Healthcare Life Science) antibodies, which were used as primary and secondary antibodies, respectively. Blots were incubated with ECL Western Blotting Detection Reagent (GE Healthcare Life Science), visualized using a Luminescent Image Analyzer LAS-4000 system (Fujifilm, Tokyo, Japan) and quantified with Multi Gauge software version 3.1 (Fujifilm).

### Immunohistochemical analysis

Mouse heart tissue samples were fixed in 4% paraformaldehyde for 24 h and embedded in paraffin. Blocks were sectioned into 4-μm-thick sections, air-dried, and deparaffinized. For wheat germ agglutinin (WGA) labeling, sections were incubated 1 h with 5 mg/ml Alexa Fluor 594-conjugated WGA (W11262, Invitrogen, Waltham, MA, USA) at room temperature. After washing, sections were cover-slipped with a water-soluble antifading mounting medium containing 4’,6-diamidino-2-phenylindole (DAPI) (Vibrance Antifade Mounting Medium with DAPI, H-1800, Vector Laboratories, Newark, CA, USA). Images were obtained and analyzed by a BZ-X710 microscope (Keyence, Osaka, Japan).

### LC-MS/MS analysis

EGT in tamogi mushroom powder (5 mg) was extracted with 1 ml of water: methanol (1:3). After removal of fat-soluble substances from the extract using a solid-phase extraction column (Discovery DSC-18; Merck), the purified solution containing EGT was dried and redissolved in 0.1% formic acid solution before LC-MS analysis. LC-MS analysis was carried out using a hybrid quadrupole-Orbitrap mass spectrometer (Q Exactive; Thermo Fischer Scientific) coupled with an Ultimate 3000 system (Thermo Fischer Scientific). The flow rate was 200 µL/min at 40 °C using a Discovery HS F5 column (150 × 2.1 mm i.d., particle size 3.0 µm; Chemicals Merck).

### Statistics and reproducibility

Sample size and information relevant to statistical tests are reported in figure legends. No statistical methods were used to determine sample size, but sample sizes were determined based on previous reports. No exclusion/inclusion criteria were applied to mice used in this study. Group allocation and outcome assessment were performed in a blinded manner. All values were reported as the mean ± SD. Data were assessed with two-group comparisons of variables using a two-sided unpaired Student’s *t* test. ANOVA data analysis was performed using GraphPad Prism software (version 7.03, GraphPad Software). For all statistical analyses, a value of *P* < 0.05 was considered statistically significant.

## Supplementary information

Supplementary Figures and Tables

Supplementary Video

## Data Availability

The datasets generated during and/or analyzed during the current study are available from the corresponding author on reasonable request.
