# Author Correction: Long-term intake of Tamogi-take mushroom (*Pleurotus cornucopiae*) mitigates age-related cardiovascular dysfunction and extends healthy life expectancy

**DOI:** 10.1038/s41514-025-00196-2

**Published:** 2025-02-19

**Authors:** Michio Sato, Daisuke Torigoe, Yuya Kinoshita, Momoka Cyuman, Chitoku Toda, Masaru Sato, Kazutaka Ikeda, Tsuyoshi Kadomatsu, Haruki Horiguchi, Jun Morinaga, Hirotaka Fukami, Taichi Sugizaki, Keishi Miyata, Ryoko Kusaba, Yusuke Okadome, Eiji Matsunaga, Koichi Node, Yuichi Oike

**Affiliations:** 1 Department of Molecular Genetics, Kumamoto University, Kumamoto, Japan; 2 Center for Metabolic Regulation of Healthy Aging (CMHA), Graduate School of Medical Sciences, Kumamoto University, Kumamoto, Japan; 3 Department of Cardiovascular Medicine, School of Medicine, Saga University, Saga, Japan; 4 Division of Kumamoto Mouse Clinic (KMC), Kumamoto University, Kumamoto, Japan; 5 Division of Laboratory Animal Science, Institute of Resource Development and Analysis (IRDA), Kumamoto University, Kumamoto, Japan; 6 Department of Neuroscience for Metabolic Control, Graduate School of Medical Sciences, Kumamoto University, Kumamoto, Japan; 7 Laboratory of Biomolecule Analysis, Department of Applied Genomics, Kazusa DNA Research Institute, Chiba, Japan; 8 Laboratory of Omics and Informatics, Department of Molecular and Chemical Life Sciences, Graduate School of Life Sciences, Tohoku University, Sendai, Japan; 9 Department of Aging and Geriatric Medicine, Graduate School of Medical Sciences, Kumamoto University, Kumamoto, Japan; 10 Department of Disease Genome Epidemiology, Center for Genomic Medicine, Graduate School of Medicine, Kyoto University, Kumamoto, Japan

**Keywords:** Cardiovascular diseases, Ageing

Correction to: *npj Aging* 10.1038/s41514-024-00191-z, published online 08 January 2025

“In this article, the affiliation details for Yuichi Oike were incorrectly given as “1,2,5,7.” The correct affiliations should be “1,2,5,9. The affiliation “Laboratory of Biomolecule Analysis, Department of Applied Genomics, Kazusa DNA Research Institute, Chiba, Japan (Affiliation 7)” was mistakenly included and has been removed, and “Department of Aging and Geriatric Medicine, Graduate School of Medical Sciences, Kumamoto University, Kumamoto, Japan (Affiliation 9)” has been added to his affiliations. The original article has been corrected to reflect these changes.”

