# Supplementary material for: Long-term intake of Tamogi-take mushroom (Pleurotus cornucopiae) mitigates age-related cardiovascular dysfunction and extends healthy life expectancy
Source: NPJ Aging. 2025 Jan 8;11(1):1. doi: 10.1038/s41514-024-00191-z (PMC11711650; doi:10.1038/s41514-024-00191-z)
Supplement: Supplementary file 1 — Supplementary Figures and Tables [file 41514_2024_191_MOESM1_ESM.pdf]

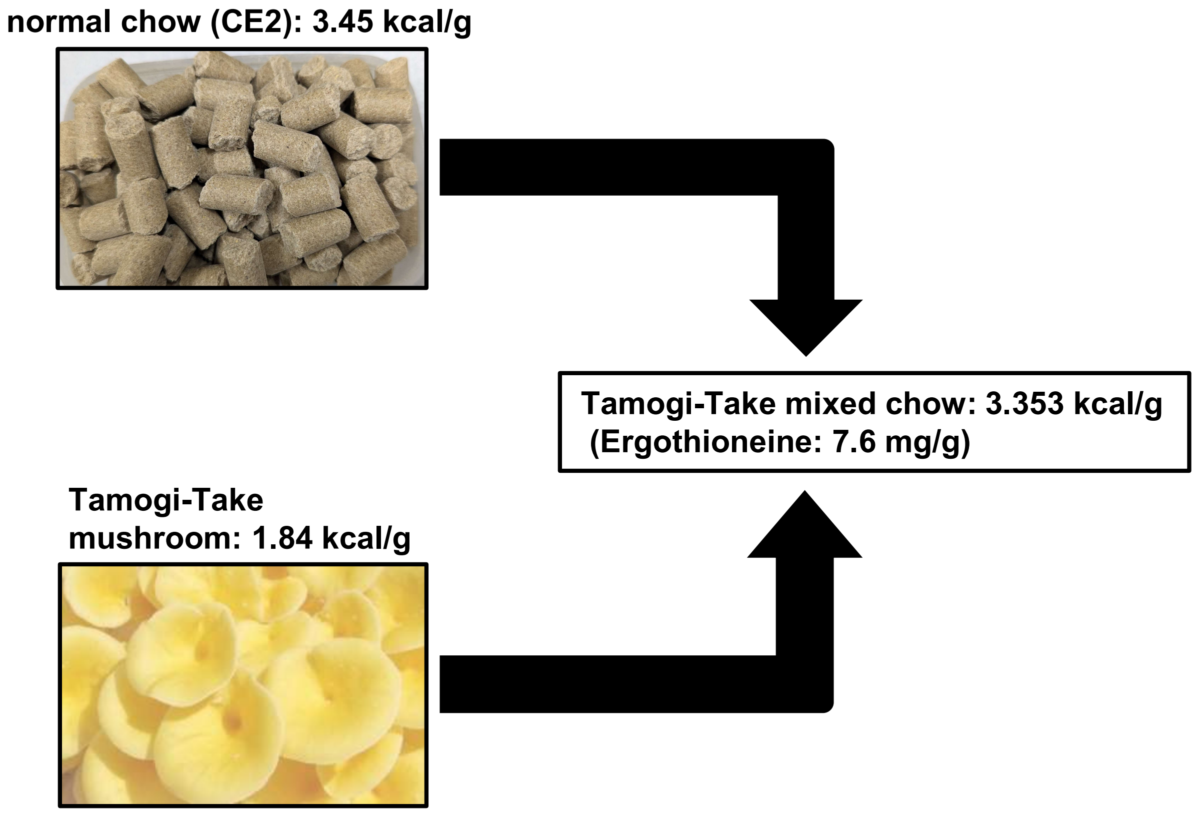

Supplementary Figure1. Preparation of Tamogi-take mushroom mixed chow.
To ensure that mice could ingest the equivalent of 70 mg/kg/day of Ergothioneine (EGT) by eating Tamogi-take mushrooms, we mixed normal chow (CE-2, see Material Methods) with Tamogi-take mushroom powder. Figure shows an example of a mixed chow, assuming that mice weigh 30g and ingest 4g of food per day. In practice, mouse weight and food intake were measured weekly, and the amount of Tamogi-take mushrooms mixed with chow was adjusted to achieve an EGT intake of 70 mg/kg/day.

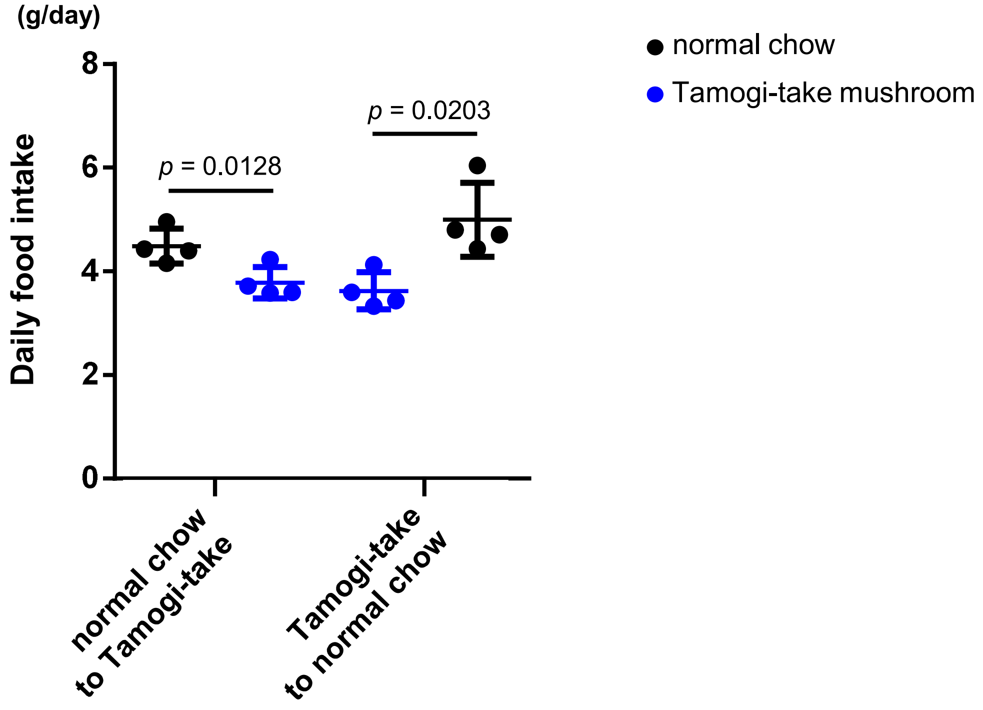

Supplementary Figure2. Administration of a Tamogi-take mushroom mixture to mice decreases food intake.

Eight-week-old male mice were divided into two groups (n = 4 per group). One was fed normal chow for 7 days followed by administration of a mixture of Tamogi-take mushrooms and chow for 4 days. The other was fed a mixture of Tamogi-take mushrooms and chow for the first 7 days followed by normal chow for 4 days. In both groups, food intake decreased during feeding with a mixed chow of Tamogi-take mushrooms.

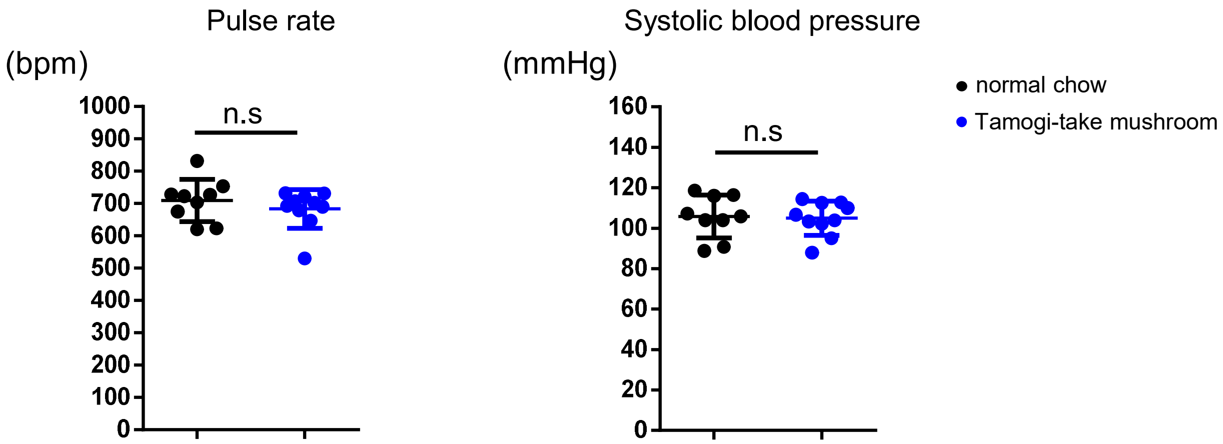

Supplementary Figure3. Long-term consumption of Tamogi-take mushrooms does not alter blood pressure or pulse rate.
Analysis of pulse rate and blood pressure in mice on fed normal chow or a Tamogi-take mushroom mixture chow from 10 weeks to 14 months of age (n = 12 per group).

a. b. c.

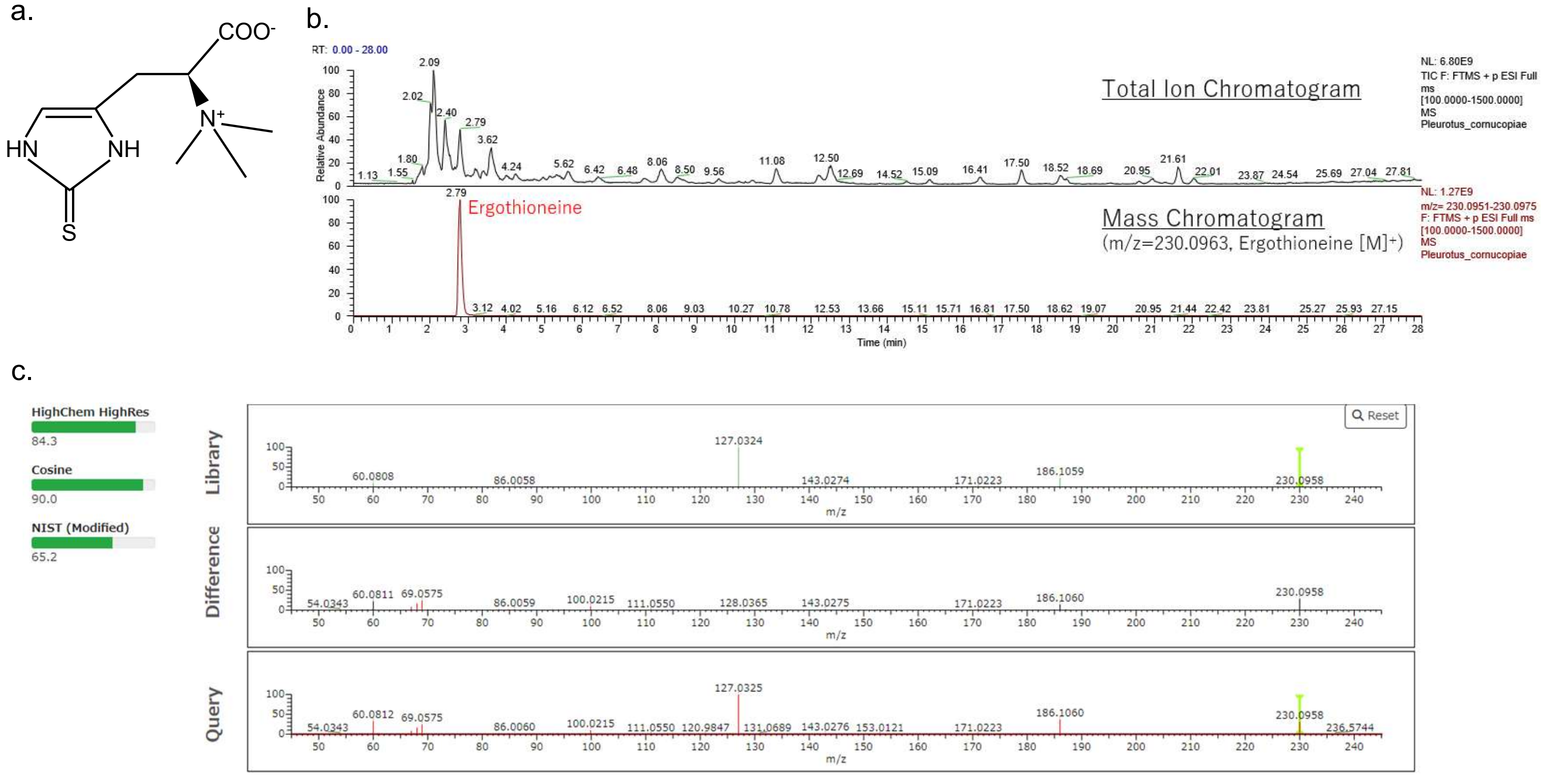

d.

Analytical test parameters	Results	Methods
Ergothioneine	7.6mg/g	High-performance liquid chromatography

Supplementary Figure4. Tamogi-take mushrooms used in this study show high Ergothioneine levels. **a.** Chemical structure of Ergothioneine (EGT). **b, c.** LC-MS/MS analysis of Tamogi-take mushroom used in this study. **d.** Amount of EGT in Tamogi-take mushrooms, based on high-performance liquid chromatography.

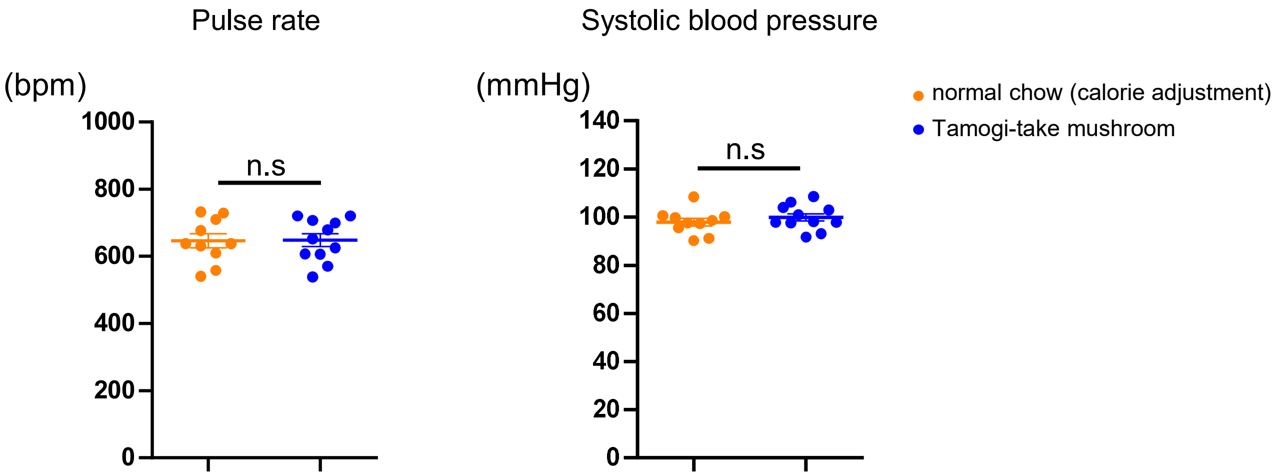

Supplementary Figure5. Treatment of mice with calorie-adjusted normal chow does not alter blood pressure or pulse rate.

Mice treated with a calorie-adjusted normal chow or Tamogi-take mushroom chow mix (n = 12 per group) between 10 weeks to 14 months of age show no changes in pulse rate or blood pressure (n = 12 per group).

Body Weight (g)	group	CE-2 (342.4kcal/100g) (g)	Tamogi-take (184kcal/100g) (g)	kcal/4g
20	normal chow	4	-	13.70
	Tamogi-take	3.82	0.18	13.41
22.5	normal chow	4	-	13.70
	Tamogi-take	3.79	0.21	13.36
25	normal chow	4	-	13.70
	Tamogi-take	3.77	0.23	13.33
27.5	normal chow	4	-	13.70
	Tamogi-take	3.75	0.25	13.30
30	normal chow	4	-	13.70
	Tamogi-take	3.72	0.28	13.25

Supplementary table1. Preparation of Tamogi-take mushroom mixed chow.
We administered a standard chow (CE-2) mixed with Tamogi-take mushroom powder. To ensure an intake of 70 mg/kg/day of ergothioneine, a compound found in Tamogi-take mushrooms, the mice were weighed weekly, as shown in this table.

Supplementary Table. 2 Primer pairs used for quantitative RT-PCR

Gene		Sequences
Rps18	Forward	TTCTGGCCAACGGTCTAGACAAC
	Reverse	CCAGTGGTCTTGGTGTGCTGA
Nppa	Forward	GAGAGACGGCAGTGCTTCTAGGC
	Reverse	CGTGACACACCACAAGGGCTTAGG
Nppb	Forward	AGGCGAGACAAGGGAGAACA
	Reverse	GGAGATCCATGCCGCAGA
Myh7	Forward	CGGACCTTGGAAGACCAGAT
	Reverse	GACAGCTCCCCATTCTCTGT
Collagen1	Forward	GAGCGGAGAGTACTGGATCGA
	Reverse	CTGACCTGTCTCCATGTTGCA
Ctgf	Forward	CAAAGCAGCTGCAAATACCA
	Reverse	GGCCAAATGTGTCTTCCAGT
PomC	Forward	GAGGCCACTGAACATCTTTGTC
	Reverse	GCAGAGGCAAACAAGATTGG
Crh	Forward	AGG GAG GAG AAG AGA GCG CCC C
	Reverse	TGC AAG GCA GGC AGG ACG AC
Npy	Forward	CTA CTC CGC TCT GCG ACA CT
	Reverse	AGT GTC TCA GGG CTG GAT CTC
Agrp	Forward	CGG CCA CGA ACC TCT GTA G
	Reverse	CTC ATC CCC TGC CTT TGC
Mc4r	Forward	CAG GCA CAG GGA CCA TCC GC
	Reverse	AAC GGG GCC CAG CAG ACA AC
Nrf-a	Forward	AAGCCTGTAGCCCACGTCGTA
	Reverse	GGCACCACTAGTTGGTTGTCTTTG
Il-1b	Forward	TCCAGGATGAGGACATGAGCAC
	Reverse	GAACGTCACACACCAGCAGGTTA
Cxcl1	Forward	CCG AAG TCA TAG CCA CAC TCA A
	Reverse	GCA GTC TGT CTT CTT TCT CCG TTA C

Gene		Sequences
Cxcl2	Forward	CTC CTT TCC AGG TCA GTT AGC
	Reverse	CAG AAG TCA TAG CCA CTC TCA A
Cx3cl	Forward	CCCTCGCCGCTCGCGTGGCTGCTGC
	Reverse	AATGGCACGCTTGCCGCAGGACTCC
Cxcl10	Forward	CTGCCTCATCCTGCTGGGTCT
	Reverse	TTAAGGAGCCCTTTTAGACC
Cdkn1a (p21)	Forward	TCAGAGCCACAGGCACCAT
	Reverse	TCCACGGGACCGAAGAGA
Trp53(P53)	Forward	GCCGATAGGTCGTTTCTTCC
	Reverse	ATCCGACTGTGACTCCTCCA
p19	Forward	GTTCTTGGTCACTGTGAGGATTCAG
	Reverse	CCATCATCATCACCTGGTCCAG
Cdkn2a (p16)	Forward	TGT TGA GGC TAG AGA GGA TCT TG
	Reverse	CGA ATC TGC ACC GTA GTT GAG C
